# Enhanced phosphorylation of T153 in soluble tau is a defining biochemical feature of the A152T tau risk variant

**DOI:** 10.1186/s40478-019-0661-2

**Published:** 2019-01-23

**Authors:** Yari Carlomagno, Dah-eun Chloe Chung, Mei Yue, Aishe Kurti, Nicole M. Avendano, Monica Castanedes-Casey, Kelly M. Hinkle, Karen Jansen-West, Lillian M. Daughrity, Jimei Tong, Virginia Phillips, Rosa Rademakers, Michael DeTure, John D. Fryer, Dennis W. Dickson, Leonard Petrucelli, Casey Cook

**Affiliations:** 1 Department of Neuroscience, Mayo Clinic Jacksonville, 4500 San Pablo Road, Jacksonville, FL 32224 USA; 2 Neurobiology of Disease Graduate Program, Mayo Clinic Graduate School of Biomedical Sciences, 4500 San Pablo Road, Jacksonville, FL 32224 USA

**Keywords:** Tauopathy, A152T, Phosphorylation, Solubility, Risk factor, Neuropathology

## Abstract

Pathogenic mutations in the tau gene (microtubule associated protein tau, *MAPT*) are linked to the onset of tauopathy, but the A152T variant is unique in acting as a risk factor for a range of disorders including Alzheimer’s disease (AD), progressive supranuclear palsy (PSP), corticobasal degeneration (CBD), and dementia with Lewy bodies (DLB). In order to provide insight into the mechanism by which A152T modulates disease risk, we developed a novel mouse model utilizing somatic brain transgenesis with adeno-associated virus (AAV) to drive tau expression in vivo, and validated the model by confirming the distinct biochemical features of A152T tau in postmortem brain tissue from human carriers. Specifically, Tau^A152T^-AAV mice exhibited increased tau phosphorylation that unlike animals expressing the pathogenic P301L mutation remained localized to the soluble fraction. To investigate the possibility that the A152T variant might alter the phosphorylation state of tau on T152 or the neighboring T153 residue, we generated a novel antibody that revealed significant accumulation of soluble tau species that were hyperphosphorylated on T153 (pT153) in Tau^A152T^-AAV mice, which were absent the soluble fraction of Tau^P301L^-AAV mice. Providing new insight into the role of A152T in modifying risk of tauopathy, as well as validating the Tau^A152T^-AAV model, we demonstrate that the presence of soluble pT153-positive tau species in human postmortem brain tissue differentiates A152T carriers from noncarriers, independent of disease classification. These results implicate both phosphorylation of T153 and an altered solubility profile in the mechanism by which A152T modulates disease risk.

**Electronic supplementary material:**

The online version of this article (10.1186/s40478-019-0661-2) contains supplementary material, which is available to authorized users.

## Introduction

Aggregation of the tau protein is a neuropathological hallmark of several neurodegenerative disorders classified as tauopathies, including frontotemporal dementia and parkinsonism linked to chromosome 17 (FTDP-17), corticobasal degeneration (CBD), progressive supranuclear palsy (PSP), Pick’s disease (PiD), and Alzheimer’s disease (AD). While mutations in the tau gene (MAPT) are known to cause primary tauopathies, no MAPT mutations were linked to AD until the discovery of the A152T mutation, which acts as a risk factor for AD [9]. In addition to modulating risk for AD, the A152T tau mutation also influences risk for dementia with Lewy bodies (DLB) [17] and the spectrum of frontotemporal dementia disorders, including PSP and CBD [9]. As such, understanding how the A152T mutation increases disease risk and identifying new genetic modifiers that impact the resulting phenotype in A152T mutation carriers could provide significant insight into the pathogenic role of tau in neurodegeneration, as well as uncover new strategies to increase resilience to tau toxicity.

In order to evaluate how the phenotype driven by A152T expression compares to the well-characterized phenotype that defines mice expressing the pathogenic P301L mutation, we used somatic brain transgenesis (SBT) to deliver adeno-associated virus (AAV) encoding either A152T or P301L mutant tau. At 3 months of age, Tau^A152T^-AAV mice exhibited increased accumulation of hyperphosphorylated tau that remained soluble, while hyperphosphorylated tau species partitioned to the insoluble fraction in Tau^P301L^-AAV mice. In addition to biochemical and neuropathological differences, we also observed a distinct behavioral phenotype in Tau^A152T^-AAV mice that included motor impairment. Most strikingly, following the development and characterization of a novel antibody, we demonstrate that the presence of the A152T variant in mice and human carriers leads to the accumulation of soluble tau species that are hyperphosphorylated on the neighboring T153 residue. Therefore the aberrant accumulation of soluble, hyperphosphorylated-T153 (pT153) tau is characteristic of A152T carriers, and may represent the underlying mechanism by which the A152T variant modulates disease risk. In addition, given that P301L and A152T differentially impact the biochemical and neuropathological profile of tau, this study has important implications for the utilization of pathogenic tau mutations that are not associated with AD to model Alzheimer’s tauopathy.

## Materials and methods

### Antibodies and reagents

Anti-IBA1 was purchased from Wako Chemicals (019–19,741, Richmond, VA), anti-GFAP was purchased from Biogenex (PU020-UP, Fremont, CA), anti-NeuN was purchased from Millipore (MAB377, Burlington, MA), biotinylated HT7 was purchased from Fisher Scientific (MN1000B, Waltham, MA), and anti-GAPDH was purchased from Meridian Life Science, Inc. (Memphis, TN). PHF1 (pS396/S404), CP13 (pS202) and MC1 (conformational epitope) were kindly provided by Dr. Peter Davies (Feinstein Institute for Medical Research, North Shore LIJ Health Care System). 12E8 (pS262/S356) was provided by Dr. Peter Seubert (previously at Elan Pharmaceuticals, San Francisco, CA). Tau 1 (dephosphorylated S195, 198, 199 and 202) was kindly provided by Dr. Nick Kanaan (Michigan State University, Grand Rapids, MI). E1 (human-specific tau antibody) was generated by our group against amino acid residues 19–33 within exon 1 of human tau [7, 10, 20]. The pT153 antibody was generated by immunizing a rabbit with a synthetic peptide encompassing residues 147–158 with the A152T variant and phosphorylation at T153 (GKTKIT[pT]PRGAA). Secondary antibodies were obtained from Jackson ImmunoResearch Laboratories, Inc. (West Grove, PA).

### Viral vector construction and AAV production

Following cloning GFP, V5-tagged Tau^A152T^, and V5-tagged Tau^P301L^ expression plasmids into an AAV vector, all constructs were sequence-verified using ABI3730 with Big Dye chemistry (Applied Biosystems, Foster City, CA). AAV vectors expressing GFP, Tau^A152T^, and Tau^P301L^ under the control of the cytomegalovirus enhancer/chicken β-actin promoter, as well as a woodchuck post-transcriptional regulatory element and the bovine growth hormone polyA, were cotransfected with AAV helper plasmids into HEK293T cells. Cells were harvested and lysed in the presence of 0.5% sodium deoxycholate and 50 U/ml Benzonase (Sigma, St. Louis, MO) by freeze thawing 48 h post-transfection, and the virus was isolated using a discontinuous iodixanol gradient. Quantitative PCR was used to measure the genomic titer of each virus.

### Intracerebroventricular injections

All animal procedures were approved by the Mayo Institutional Animal Care and Use Committee (IACUC) and are in accordance with the National Institutes of Health Guide for the Care and Use of Laboratory Animals (NIH Publications No. 80–23, revised 1996). Mouse pups were injected with either Tau^A152T^-AAV, Tau^P301L^-AAV or GFP-AAV intracerebroventricularly (ICV) on postnatal day 0 (2.7E+ 10 viral particles/ventricle; 2uL/ventricle). ICV injections were performed as described [4]. Briefly, newborn mice were cryoanesthetized and placed on a cold metal plate. A 10uL Hamilton syringe with a 30 gauge needle was used to pierce the skull just posterior to bregma and 2 mm lateral to the midline, and 2uL of AAV was injected into each of the lateral ventricles. Behavioral testing was performed at 3 months of age, at the conclusion of which animals were euthanized by CO2 inhalation. The brain was immediately removed and bisected sagittally, with one hemi-brain drop-fixed in 10% neutral buffered formalin (Fisher Scientific, Waltham, MA) and incubated overnight at 4 °C for histology, while the other half was frozen for RNA and biochemical analysis.

### Histology and immunohistochemistry

The formalin-fixed half brain was embedded in paraffin wax, sectioned in a sagittal plane at a thickness of 5 μm, and mounted on glass slides. The tissue sections were deparaffinized in xylene and rehydrated in a graded series of alcohols. Antigen retrieval was performed by steaming in distilled water for 30 min, and endogenous peroxidase activity was blocked by incubation in 0.03% hydrogen peroxide. Sections were then immunostained using the DAKO Autostainer (DAKO North America, Carpinteria, CA) and the DAKO EnVision + HRP system. The stained slides were then dehydrated, cover-slipped, and scanned with the Aperio Slide Scanner (Aperio, Vista, CA). Quantitative analysis of CP13, GFAP, and IBA1 [*n* = 11 Tau^P301L^-AAV, *n* = 12 Tau^A152T^-AAV] immunoreactivity was performed using a custom-designed color deconvolution algorithm and ImageScope software (Aperio, Vista, CA). As previously described [15], the algorithm was designed to measure the optical density of the brown chromagen as a percentage of burden within an annotated region of interest. The assessment of neuronal loss was performed by quantifying NeuN-positive nuclei per mm^2^ of cortex [n = 11 GFP-AAV, n = 12 Tau^A152T^-AAV].

### Preparation of mouse brain homogenates and biochemical analysis

Half brains were weighed and homogenized in 5x volume of TE buffer (50 mM Tris base [pH 7.4], 50 mM NaCl, 1 mM EDTA, 1 mM PMSF, 1x protease and phosphatase inhibitors). For RNA extraction 70uL of homogenate was added to 210uL Trizol LS (Life Technologies, Carlsbad, CA), and the mixture frozen at -80 °C until extraction was performed, described below. For the sequential extraction method described [22], 250uL of homogenate was added to 250uL of TBS (50 mM Tris [pH 8], 274 mM NaCl, 5 mM KCl, 1 mM PMSF, and a protease and phosphatase inhibitor cocktail), and the sample ultracentrifuged at 150,000 g for 15 min at 4 °C. The supernatant (S1 fraction) was transferred to a new Eppendorf tube, and the pellet homogenized in 3x volume buffer (10 mM Tris [pH 7.4], 0.8 M NaCl, 10% sucrose, 1 mM EGTA, 1 mM PMSF) and subsequently ultracentrifuged at 150,000 g for 15 min at 4 °C. The pellet was discarded, and the supernatant incubated with sarkosyl at a final concentration of 1% for 1 h at 37 °C. Following this incubation, the samples were ultracentrifuged at 150,000 g for 30 min at 4 °C, the supernatant (S2 fraction) transferred to a new Eppendorf tube, and the pellet (P3 fraction) resuspended in TE buffer (10 mM Tris [pH 8], 1 mM EDTA). A BCA protein assay (Pierce Biotechnology, Rockford, IL) was performed on the S1 and S2 fractions to determine protein concentration. Protein (10-20 μg) from each sample was diluted in dH_2_O, 2x Tris-glycine SDS sample buffer (Life Technologies), and 5% beta-mercaptoethanol (Sigma Aldrich), and heat-denatured for 5 min at 95 °C. Samples were run on 10% or 4–20% SDS-PAGE Tris-glycine gels (Life Technologies), and transferred to PVDF membrane (Millipore). Membranes were blocked in 5% non-fat dry milk in TBS/0.1% Triton-X-100, and incubated overnight in primary antibody diluted in 5% milk in TBS/0.1% Triton-X-100 rocking at 4 °C. Membranes were incubated in HRP-conjugated secondary antibodies (1:5000; Jackson ImmunoResearch) for 1 h at room temperature, and detected by ECL (Thermo Fisher Scientific, Rockford, IL). Bands were quantified using Scion Image by analyzing pixel density, and protein levels were normalized to the protein loading control. MesoScale Discovery (MSD) immunoassays were also performed as described [8] to measure tau species in the S1 and S2 fractions using the human tau specific E1 antibody as the capture, and either biotinylated-HT7, Tau 5, PHF1 or MC1 as the detection antibody. To measure Tau1-positive human tau, Tau1 was used as the capture antibody, and E1 used to detect. Group sizes for all biochemical studies included *n* = 11 Tau^P301L^-AAV and *n* = 12 Tau^A152T^-AAV mice.

### Human postmortem tissue

Human postmortem brain tissue was provided by the brain bank at Mayo Clinic Jacksonville. For these studies, frontal cortex was used from A152T carriers and noncarriers matched for pathological diagnosis, Braak stage, age and gender (see Table 1). Tissue (approximately 250 mg) was homogenized in 6x volume of buffer (50 mM Tris [pH 8], 274 mM NaCl, 5 mM KCl, 1 mM PMSF, and a protease and phosphatase inhibitor cocktail), and the sample ultracentrifuged at 150,000 g for 15 min at 4 °C. The supernatant (S1 fraction) was transferred to a new Eppendorf tube, and a BCA protein assay performed to determine protein concentration. Thirty micrograms of protein from each sample was diluted in dH_2_O, 2x Tris-glycine SDS sample buffer and 5% beta-mercaptoethanol, and immunoblotting performed as described above.

**Table 1:** Information about human postmortem tissue samples

A152T carriers			Noncarriers		
Code	Braak	Age; Gender	Code	Braak	Age; Gender
ADv1	5	81; F	AD1	6	80; F
ADv2	6	96; M	AD2	5.5	91; M
ADv3	6	86; M	AD3	5	84; M
ADv4	4.5	80; F	AD4	5	80; F
ADv5	6	91; F	AD5	6	83; F
ADv6	5	67; M	AD6	6	61; M
PSPv1	2	67; M	PSP1	1	69; M
PSPv2	3	74; F	PSP2	3	74; M
PSPv3	1	81; F	PSP3	1	80; F
PSPv4	3	77; M	PSP4	2.5	76; M
PSPv5	3	77; F	PSP5	3.5	81; F
LBDv1	3	75; M	LBD1	3.5	72; M
LBDv2	2.5	72; M	LBD2	0	72; M
CBDv1	3	68; F	CBD1	3	67; F
fMNDv1 (C9+)	0	62; F	fMND1 (C9+)	2	55; F

*AD* Alzheimer’s disease, *PSP* Progressive supranuclear palsy, *LBD* Lewy body dementia, *CBD* Corticobasal degeneration, *fMND (C9+)* Frontotemporal lobar degeneration with motor neuron disease associated with C9ORF72 mutation

### RNA preparation and qRT-PCR

Total RNA was isolated from brain tissue using the Direct-zol RNA Miniprep kit (Zymo Research, Irvine, CA) according to manufacturer’s instructions with in-column DNase I treatment. Random-primed reverse transcription was performed using the High capacity cDNA reverse transcription kit according to manufacturer protocols (Applied Biosystems, Foster City, CA). cDNA was diluted 1:40 and added to a reaction mix (5 μL final volume) containing 100 nM gene-specific primers and SYBR GreenER qPCR supermix universal (Thermo Fisher Scientific, Rockford, IL). All samples were run in triplicate and were analyzed on an ABI 7900 HT Fast Real Time PCR instrument (Applied Biosystems - Life Technologies). Relative gene expression was normalized to GAPDH controls and assessed using the 2^-ΔΔCT^ method. Primer sequences are as follows (5′ to 3′): *Gapdh* F: CTGCACCACCAACTGCTTAG, *Gapdh* R: ACAGTCTTCTGGGTGGCA GT, *Aif1 (Iba1)* F: GGATTTGCAGGGAGGAAAAG *Aif1* (Iba1) R: TGGGATCATCGAGGAATTG, *Gfap* F: GGAGAGGGACAACTTTGCAC, *Gfap* R: AGCCTCAGGTTGGTTTCATC, human-specific *MAPT* F: CTCCAAAATCAGGGGATCGC, human-specific *MAPT* R: CCTTGCTCAGGTCAACTGGT [1, 13]. Group sizes for qRT-PCR analysis included *n* = 16 GFP-AAV, *n* = 11 Tau^P301L^-AAV, and *n* = 12 Tau^A152T^-AAV mice.

### Behavioral assessment

The battery of behavioral tasks utilized in the current study includes: open field assay (OFA), elevated plus maze (EPM) test, contextual and cued fear conditioning, and rotarod. For each test, mice were acclimated to the room of testing for 1 h, and all tests were performed during the first half of the light cycle (with the exception of cued fear conditioning) on consecutive days, as described [6]. Behavioral equipment was cleaned with 30% ethanol between animals, and mice were returned to their home cage and holding room at the conclusion of each test. Group sizes for behavioral testing included n = 16 GFP-AAV, *n* = 20 Tau^P301L^-AAV, and *n* = 12 Tau^A152T^-AAV.

#### Open-field assay

Mice were allowed to roam freely around an open-field arena (40 × 40 × 30 cm, W x L x H) for 15 min, and an overhead camera was used to track movement with AnyMaze software (Stoelting Co., Wood Dale, IL). Multiple measures were analyzed, including total distance traveled, average speed, time mobile, and distance traveled in the “center” zone (20 × 20 cm).

#### Elevated plus maze test

The elevated plus maze is elevated 50 cm from the floor, and consists of four arms (50 × 10 cm) with two of the arms open, and two arms enclosed with roofless gray walls (35 × 15 cm, L x H). Mice were placed in the center of the maze facing an open arm, and their behavior was tracked for 5 minutes with an overhead camera and AnyMaze software.

#### Contextual and cued fear conditioning test

A sound-proof chamber with a grid floor capable of delivering an electric shock was used for this test, with time spent freezing measured by an overhead camera and FreezeFrame software (Actimetrics, Wilmette, IL). Baseline freezing behavior was recorded by placing mice in the chamber and leaving them undisturbed for 2 min, following which a conditioned stimulus (CS; 80-dB white noise) was presented for 30 s. In the last 2 s of the CS, mice received a mild foot shock (0.5 mA), which served as the unconditioned stimulus (US). An additional CS-US pair was presented 1 min later, and the mouse was removed and returned to its home cage 30 s after the second CS-US pair. Twenty-four hours later, the contextual fear conditioning test was performed in which each mouse was returned to the test chamber and freezing behavior was recorded for 5 min. Mice were then returned to their home cage and placed in a different room than previously tested with reduced lighting conditions, and allowed to acclimate for 1 hour. For the cued fear conditioning test, environmental and contextual cues were changed by: cleaning testing chambers with 30% isopropyl alcohol instead of 30% ethanol; replacing white house lights with red house lights; placing a colored plastic triangular insert in the chamber to alter its shape and spatial cues; covering the wire grid floor with opaque plastic; and altering the smell in the chamber and testing room with vanilla extract. The mice were then placed in the chamber and left undisturbed for 3 min, at which time the auditory CS was presented and freezing was recorded for another 3 min. Baseline freezing behavior obtained during training was subtracted from both context and cued tests.

#### Rotarod

Mice are placed on an accelerating rotarod apparatus for a total of 4 trials per day, with a 30–60-min interval between trials, for four consecutive days. Each trial runs for the maximum duration of 5 min, during which the rod gradually accelerates from 4 to 40 rpm. The amount of time for each mouse to fall from the rod (approximately 6 in. from the ground) is recorded for each trial.

### Statistical analyses

To determine whether differences between GFP-AAV, Tau^P301L^-AAV, and Tau^A152T^-AAV animals were statistically significant, differences between groups were assessed using 1-way ANOVA followed by a Tukey’s posthoc test for multiple comparisons. To evaluate the statistical significance between Tau^P301L^-AAV and Tau^A152T^-AAV mice, unpaired two-tailed *t* tests were performed. All statistical analyses were performed in GraphPad Prism, and are presented as mean +/− SEM, with *p* < 0.05 considered statistically significant.

## Results

### Tau deposition differs in mice expressing the pathogenic P301L mutation and the A152T risk variant

**Fig. 1:**
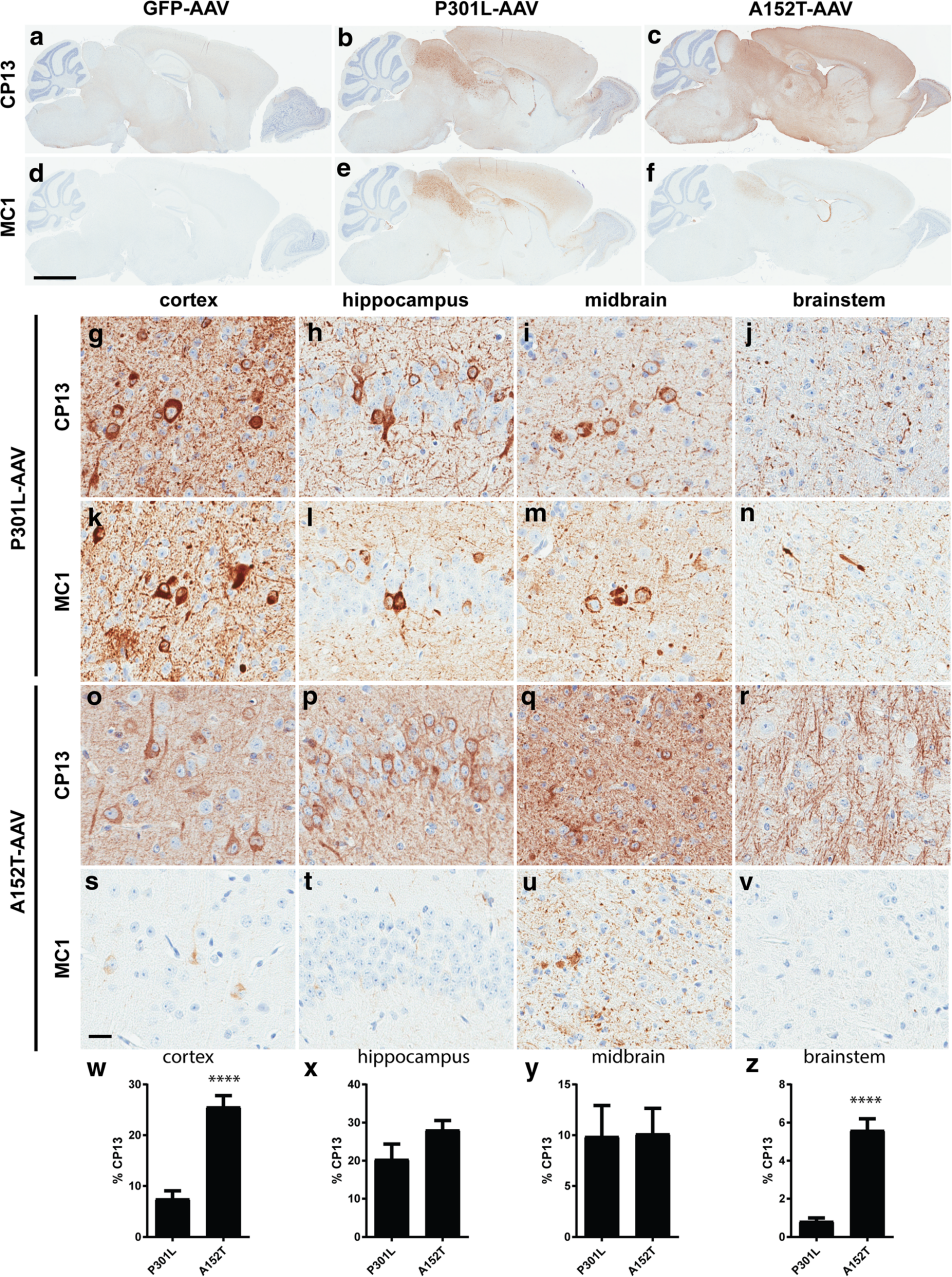
Striking differences in the pattern of tau accumulation in P301L and A152T-expressing animals. **a**-**f** Representative low-power images comparing the distribution and intensity of CP13 and MC1 immunoreactivity in GFP-AAV (**a** and **d**), Tau^P301L^-AAV (**b** and **e**), and Tau^A152T^-AAV mice (**c** and **f**). **g**-**v** High-power images revealing differences in the deposition of CP13 and MC1-positive tau species between Tau^P301L^-AAV (**g**-**n**) and Tau^A152T^-AAV (**o**-**v**) mice in the cortex (**g**, **k**, **o** and **s**), hippocampus (**h**, **l**, **p** and **t**), midbrain (**i**, **m**, **q** and **u**) and brainstem (**j**, **n**, **r** and **v**). **w** Quantitative analysis of CP13 immunoreactivity was performed in cortex (*t* = 6.64, *p* < 0.0001); **x** hippocampus (*t* = 1.6, *p* = 0.12); **y** midbrain (*t* = 0.07, *p* = 0.95); and **z** brainstem (*t* = 7.36, *p* < 0.0001). Scale bar in (**a**-**f**) equals 2 mm; scale bar in (**g**-**v**) equals 20 μm. ****p < 0.0001

Taking advantage of the versatile model of tauopathy we recently developed [8], we generated Tau^A152T^-AAV and utilized somatic brain transgenesis (SBT) on postnatal day 0 (P0) to compare expression of Tau^A152T^-AAVand Tau^P301L^-AAV in the brain. At 3 months of age, strong immunoreactivity for the phospho-tau epitope CP13 (pS202) was detected in both Tau^P301L^-AAV and Tau^A152T^-AAV -injected mice, although the pattern of CP13-positivity was very different. Specifically, CP13 immunoreactivity in Tau^P301L^-AAVmice exhibited an intense and punctate labeling pattern with abundant deposition in the cell soma (Fig. 1b, g-j), while CP13 immunolabeling was very diffuse with significant labeling of the neuropil in Tau^A152T^-AAV mice (Fig. 1c, o-r). Regionally, the accumulation of CP13-positive tau in Tau^A152T^-AAV mice was most significantly increased in the cortex and brainstem relative to Tau^P301L^-AAV mice (Fig. 1w, z), with CP13 levels in the hippocampus and midbrain relatively equal between the two models (Fig. 1x-y). Striking differences were also noted with the MC1 epitope, which detects an abnormal conformational change that occurs early in neurofibrillary tangle (NFT) formation [16, 24]. In particular, minimal MC1 immunoreactivity was detected in Tau^A152T^-AAV mice (Fig. 1f, s-v) in comparison to the significant accumulation in Tau^P301L^-AAV mice (Fig. 1e, k-n).

**Fig. 2:**
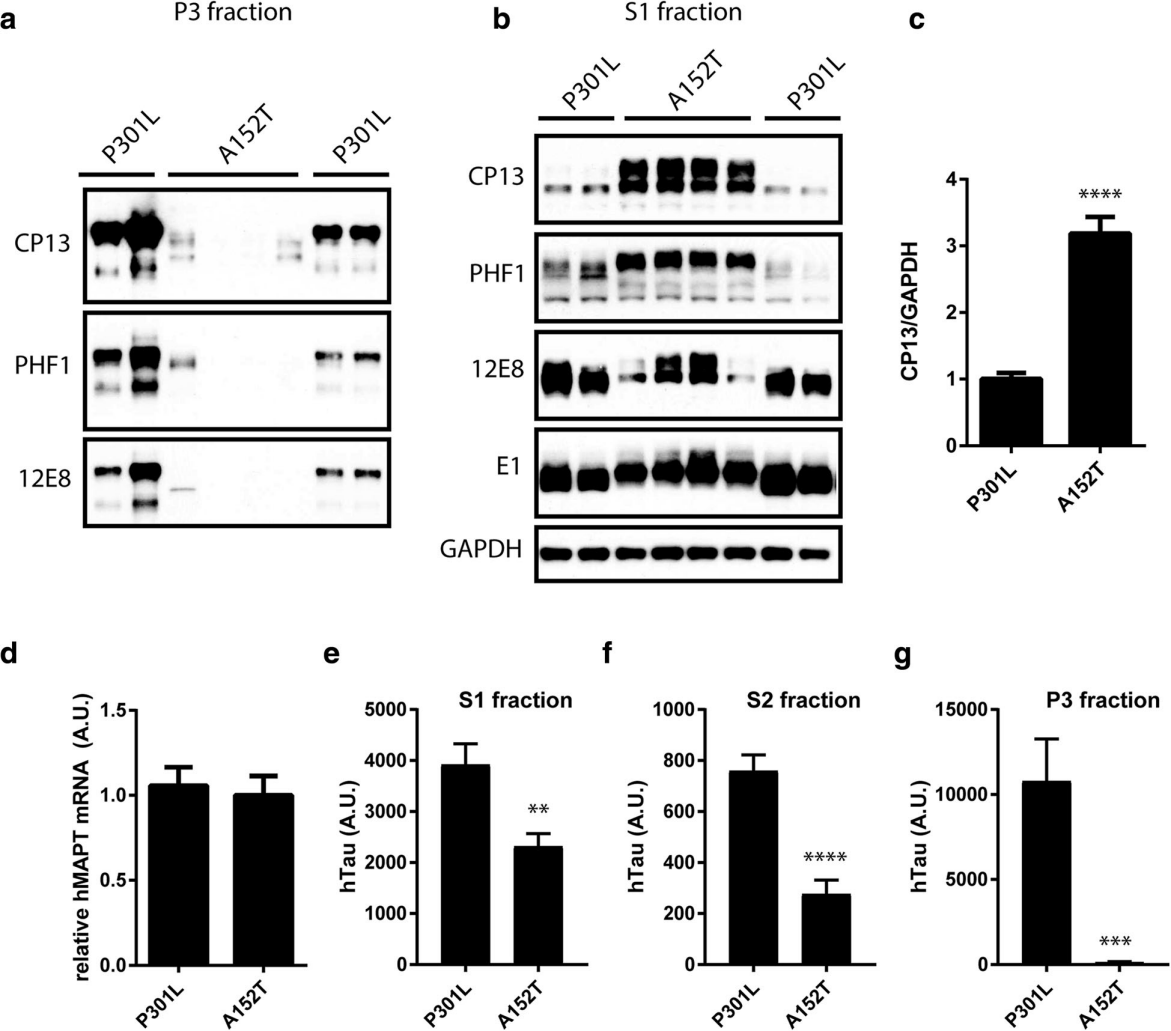
Mutant A152T tau accumulates in the soluble fraction. **a** and **b** Biochemical fractionation into sarkosyl-insoluble P3 fraction (**a**) and soluble S1 fraction (**b**) indicate hyperphosphorylated tau remains soluble in Tau^A152T^-AAV mice and becomes insoluble in Tau^P301L^-AAV mice. **c** Quantitation of CP13 levels in the S1 fraction normalized to GAPDH as a control for protein loading (*t* = 7.86, *p* < 0.0001). **d** Human tau mRNA levels are equal between Tau^P301L^-AAV and Tau^A152T^-AAV mice (*t* = 0.35, *p* = 0.73). **e** Human tau protein levels in S1 fraction measured by MSD immunoassay are significantly lower in Tau^A152T^-AAV compared to Tau^P301L^-AAV mice (*t* = 3.382, *p* = 0.0028). **f** Human tau levels in S2 fraction are also reduced in Tau^A152T^-AAV relative to Tau^P301L^-AAV mice (*t* = 5.835, *p* < 0.0001). **g** Minimal human tau is detected in the P3 fraction in Tau^A152T^-AAV mice (*t* = 4.549, *p* = 0.0002). **p < 0.005, ***p < 0.001, ****p < 0.0001

Given the observation that P301L and A152T lead to a very different pattern of tau deposition in vivo, we then wanted to determine whether these mutations might differentially impact tau solubility. Following sequential extraction and subsequent analysis by immunoblot and immunoassay, we evaluated tau levels in the most soluble (S1) fraction, as well as the sarkosyl-soluble (S2) and sarkosyl-insoluble (P3) fractions (Fig. 2, Additional file 1: Figures S1 and S2). Consistent with our previous findings characterizing the Tau^P301L^-AAV model [8], accumulation of tau phosphorylated on multiple epitopes was detected in the P3 fraction from Tau^P301L^-AAV mice (Fig. 2a). In contrast, hyperphosphorylated tau was predominantly absent from the P3 fraction in Tau^A152T^-AAV mice (Fig. 2a), while significant deposition of hyperphosphorylated tau species was detected in soluble S1 and S2 fractions (Fig. 2b-c; Additional file 1: Figures S1 and S2). Among the phospho-epitopes evaluated, soluble CP13 and PHF1 (pS396/404) appeared to be most significantly elevated in Tau^A152T^-AAV compared to Tau^P301L^-AAV mice (Fig. 2b-c; Additional file 1: Figures S1a, d and S2), while 12E8 (pS262/356) was relatively equal (Fig. 2b; Additional file 1: Figure S1b). Consistent with increased phosphorylation, we detected a significant reduction in tau species detected with the unphosphorylated-specific antibody Tau1 in both S1 (Additional file 1: Figure S1b) and S2 fractions in Tau^A152T^-AAV mice (Additional file 1: Figure S2a-b).

To determine whether the increased accumulation of soluble hyperphosphorylated tau in Tau^A152T^-AAV compared to Tau^P301L^-AAV mice was due to a higher level of expression, we measured total human tau mRNA and protein levels. This analysis revealed that although mRNA expression levels were equal (Fig. 2d), human tau protein levels were lower in both S1 (Fig. 2e) and S2 (Fig. 2f), and largely absent from the P3 fraction (Fig. 2g) in Tau^A152T^-AAV mice. These results might indicate that A152T tau is less resistant to degradation than P301L tau, which is consistent with a recent report demonstrating that the detrimental impact of the A152T variant on tau degradation was less aggressive than the P301L mutation [3].

Finally, in agreement with the significant reduction in MC1 immunoreactivity in Tau^A152T^-AAV relative to Tau^P301L^-AAV mice (Fig. 1e-f, k-n, s-v), we only detected minimal MC1 in the S1 fraction from Tau^A152T^-AAV mice by MSD immunoassay (Additional file 1: Figure S1f). Thus based on the fact that hyperphosphorylated tau epitopes accumulate in the S1 fraction in Tau^A152T^-AAV mice and in the P3 fraction in Tau^P301L^-AAV mice, these findings indicate the A152T mutation slows aggregation of hyperphosphorylated tau possibly by inhibiting the adoption of the MC1 conformation. This is consistent with the diffuse pattern of CP13 immunolabeling and weak MC1 immunoreactivity in Tau^A152T^-AAV relative to Tau^P301L^-AAV mice, in addition to a previous report demonstrating that the A152T mutation favors tau oligomer and impedes filament formation in vitro [9].

### Mutant A152T hTau expression is associated with gliosis and neuronal loss

**Fig. 3:**
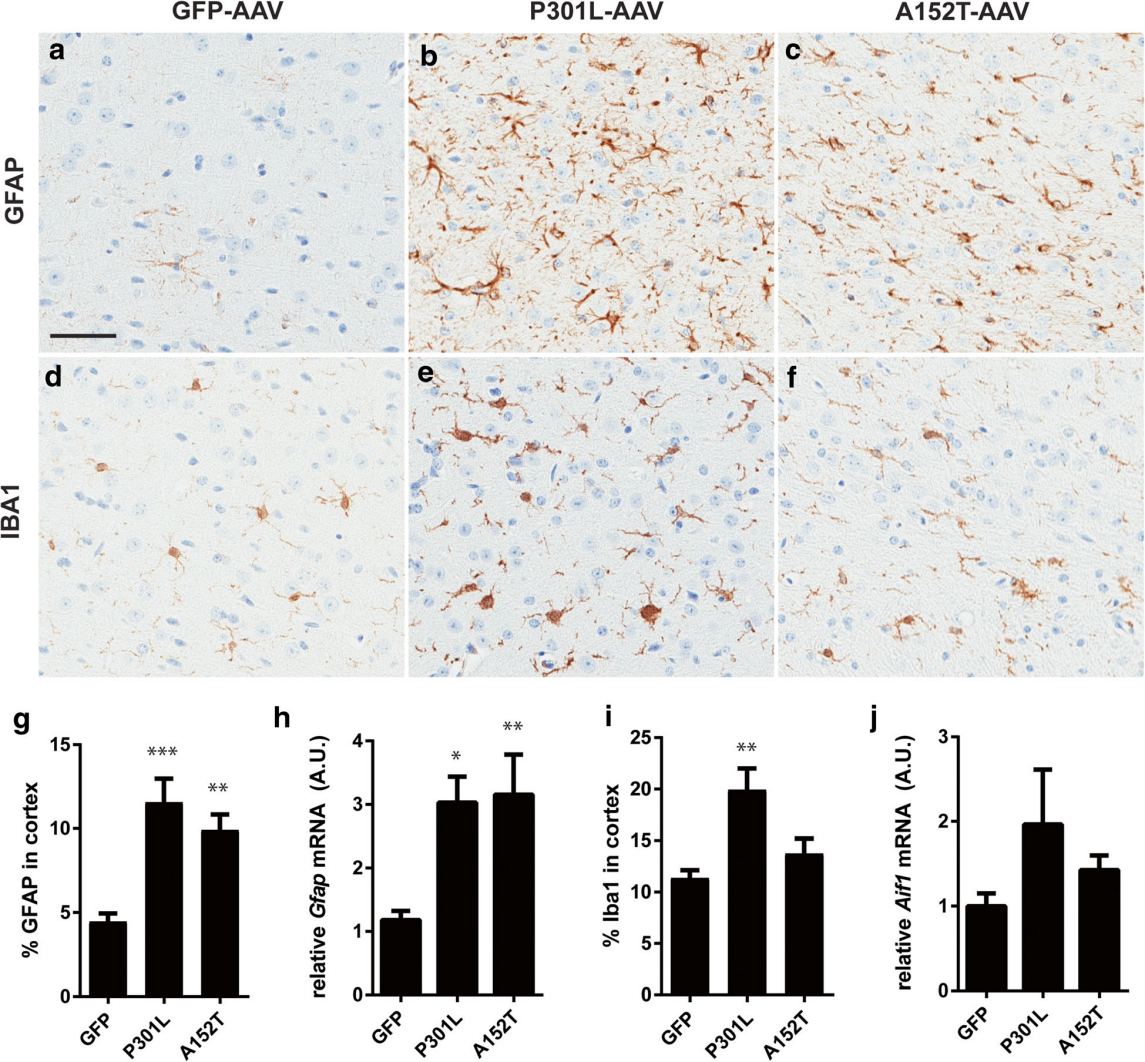
Increased astrogliosis in Tau-AAV mice. **a**-**f** Astrocytes and microglia were labeled with GFAP (**a**-**c**) and IBA1 (**d**-**f**), respectively, and representative images presented from the cortex. **g** GFAP immunoreactivity in the cortex was significantly increased in both Tau^P301L^-AAV and Tau^A152T^-AAV mice (F = 11.14, *p* = 0.0003). **h** GFAP is also significantly increased at the mRNA level in both Tau-AAV mouse models relative to GFP-AAV mice (F = 5.93, *p* = 0.0016). **i** Quantification of IBA1 immunolabeling in the cortex detected a significant increase only in Tau^P301L^-AAV mice (F = 6.96, *p* = 0.0036). **j** RT-qPCR analysis revealed no significant differences of IBA1 (*Aif1*) mRNA levels between groups (F = 2.23, *p* = 0.12). Scale bar in (**a**) equal to 50 μm. *p < 0.05, **p < 0.005, ***p < 0.001

As several inflammatory changes in the absence of neuronal loss are detected in Tau^P301L^-AAV mice at 6 months of age [8], we wanted to assess how these neurodegenerative endpoints compare with the Tau^A152T^-AAV model at 3 months. We first evaluated immunoreactivity for GFAP (Fig. 3a-c), which revealed significant astrocytosis in both Tau^P301L^-AAV and Tau^A152T^-AAV mice reflected by an increase in GFAP immunostaining in the cortex (Fig. 3g), in addition to an upregulation at the mRNA level relative to GFP-AAV mice (Fig. 3h). We also observed marked microgliosis in Tau^P301L^-AAV mice following the assessment of IBA1 immunoreactivity in the cortex (Fig. 3e, i), although mRNA levels were not significantly increased in the brain (Fig. 3j). In contrast to Tau^P301L^-AAV mice, IBA1 immunoreactivity in the cortex was not increased in Tau^A152T^-AAV relative to GFP-AAV mice (Fig. 3d, f, i). In addition, despite the absence of a significant microglial response in Tau^A152T^-AAV mice, we observed a reduction in the number of NeuN-positive nuclei in the cortex (Additional file 1: Figure S3). Collectively, these findings indicate that the accumulation of soluble, hyperphosphorylated tau species in Tau^A152T^-AAV mice is associated with astrocytosis and neuronal loss, which is consistent with other transgenic A152T mouse models [19, 23].

### Abnormal behavioral phenotype distinguishes P301L and A152T-expressing mice

**Fig. 4:**
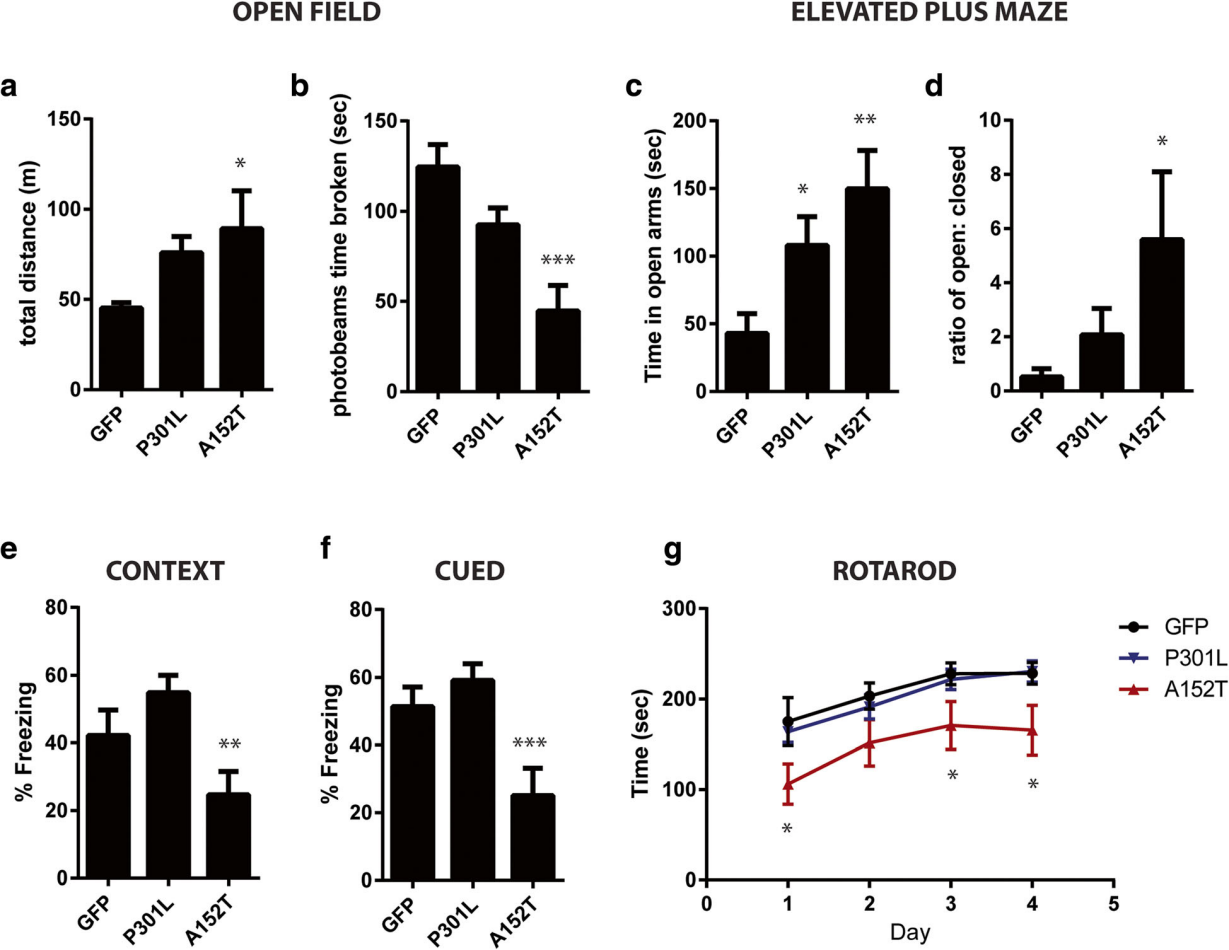
Significant behavioral abnormalities in Tau^A152T^-AAV mice. **a** and **b** Behavior in open field analysis revealed an increase in total distance traveled (**a** F = 3.3, *p* = 0.04) and decrease in time spent breaking photobeams (**b** F = 10.51, *p* = 0.0002) in Tau^A152T^-AAV compared to either GFP-AAV or Tau^P301L^-AAV mice. **c** and **d** Exploratory behavior was also evaluated in elevated plus maze (EPM), which detected a significant increase in the time Tau-AAV mice spent in open arms (**c** F = 5.71, *p* = 0.006), as well as an increase in Tau^A152T^-AAV mice in the ratio of time spent in open to closed arms (**d** F = 4, *p* = 0.026). **e** and **f** Cognitive function was assessed by fear conditioning, which demonstrated that Tau^A152T^-AAV mice are significantly impaired in both context (**e** F = 5.62, *p* = 0.0067) and cued (**f** F = 7.835, *p* = 0.0012) versions of the task. **g** Rotarod was utilized to measure motor function, exposing significant motor impairment in Tau^A152T^-AAV mice (Day 1: F = 5.18, *p* = 0.009; Day 3: F = 3.33, *p* = 0.04; Day 4: F = 4.4, *p* = 0.018). *p < 0.05, **p < 0.01, ***p < 0.001

To define the behavioral consequences of mutant P301L and A152T tau expression, we evaluated performance on several behavioral tasks. By 3 months of age, Tau^A152T^-AAV mice exhibited increased hyperactivity and decreased rearing (as indicated by time photobeams were broken) relative to GFP-AAV mice in open field analysis (Fig. 4a-b). Tau^A152T^-AAV mice also spent significantly more time in open arms of an elevated plus maze (Fig. 4c-d) indicative of disinhibition. We also observed significantly reduced freezing in Tau^A152T^-AAV mice in a contextual and cued fear conditioning testing paradigm (Fig. 4e-f), implicating the involvement of cognitive impairment in the phenotype driven by A152T expression. Although Tau^P301L^-AAV mice displayed similar aberrant behaviors at 6 months of age [8], they appeared relatively normal at 3 months of age in both open field (Fig. 4a-b) and fear conditioning (Fig. 4e-f), while increased exploration of open arms in elevated plus maze is already apparent (Fig. 4c-d). In addition to measures of anxiety and cognitive function, we also wanted to determine whether mutant tau expression would impact motor function. Intriguingly, Tau^A152T^-AAV mice were significantly impaired on the rotarod task, while Tau^P301L^-AAV and GFP-AAV mice performed equally well (Fig. 4g), indicating the appearance of motor deficits further distinguishes Tau^A152T^-AAV from Tau^P301L^-AAV mice. To evaluate the basis of motor dysfunction in the Tau^A152T^-AAV animals, we quantified tau levels in the spinal cord. While there was a slight increase in human tau expression in spinal cord of Tau^A152T^-AAV compared to Tau^P301L^-AAV mice (Additional file 1: Figure S4a-c), the amount of tau phosphorylated on the PHF1 epitope (pS396/404) was significantly increased in the spinal cord of Tau^A152T^-AAV mice (Additional file 1: Figure S4d).

### A152T variant is associated with accumulation of soluble pT153 in mice and humans

**Fig. 5:**
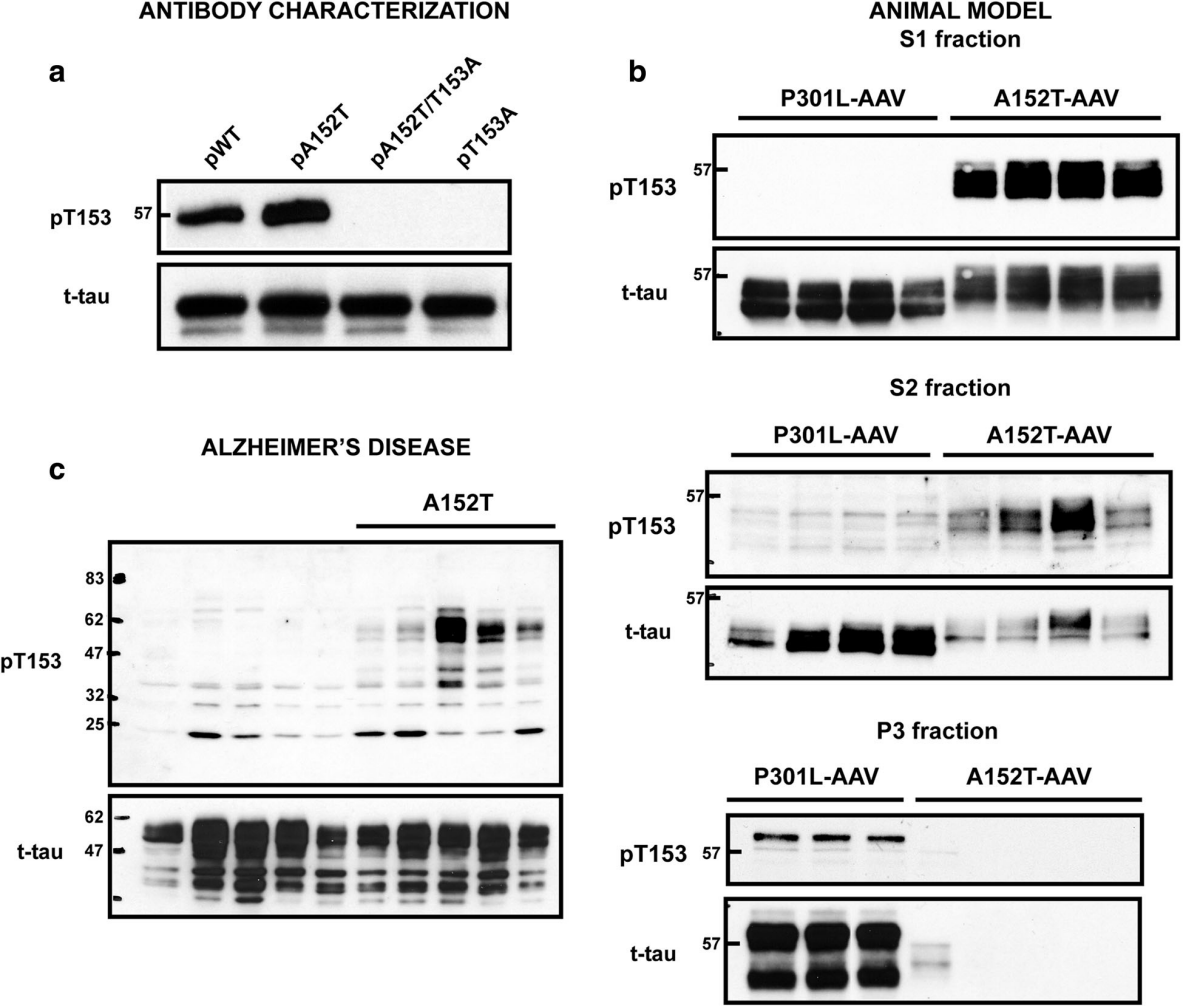
Accumulation of soluble pT153 is specific to A152T carriers. **a** Recombinant wild-type (WT) and mutant tau proteins (including A152T, A152T/T153A, and T153A) were phosphorylated in vitro with GSK3β, and subsequently evaluated by immunoblotting. 100 ng of each recombinant phosphorylated protein was loaded, followed by detection with phospho-specific antibody (pT153) and E1 (human-tau specific antibody) to confirm equal protein loading. **b** Biochemical fractionation followed by immunoblotting reveals significant accumulation of pT153 in the S1 and S2 soluble fractions in A152T-AAV mice, while pT153-positive tau is detected in the P3 fraction in P301L-AAV mice. **c** The amount of pT153 and total tau in the soluble S1 fraction from frontal cortex of A152T carriers and noncarriers with Alzheimer’s disease (AD) was evaluated by immunoblotting

To determine if the A152T variant alters the pattern of tau phosphorylation, either through introduction of a new phosphorylation site at T152 or by influencing phosphorylation of the neighboring T153, we generated a series of antibodies. Specifically, rabbits were immunized with peptides containing the A152T variant that were phosphorylated on either T152, T153, or both T152 and T153. Antibody specificity was then assessed by evaluating detection of phosphorylated wild-type (WT) and mutant recombinant tau proteins. While the antibodies generated against T152 or the dual T152/153 were excluded due to nonspecific detection of either unphosphorylated tau or phosphorylated T153A tau (data not shown), the antibody generated against phosphorylated T153 (pT153) was quite specific and detects pT153 in both WT and A152T tau (Fig. 5a). We then examined pT153 immunoreactivity in AAV-injected animals, which revealed significant accumulation of pT153 in the soluble S1 and S2 fractions in Tau^A152T^-AAV mice, while this tau species was absent from the sarkosyl-insoluble P3 fraction of A152T-expressing mice (Fig. 5b). In contrast, pT153-positive tau species were detected in the P3 sarkosyl-insoluble fraction of Tau^P301L^-AAV mice, with minimal pT153 immunoreactivity observed in soluble fractions (Fig. 5b).

To assess the relevance of this observation to human A152T carriers, we identified noncarriers that were matched as closely as possible to each A152T carrier for pathological diagnosis, Braak stage, age and gender (see Table 1). Following biochemical fractionation and subsequent immunoblotting of the soluble S1 fraction, consistent with the Tau^A152T^-AAV model we observed robust accumulation of soluble pT153-positive tau species in A152T carriers relative to noncarriers in AD (Fig. 5c), PSP (Additional file 1: Figure S5a), as well as LBD, CBD and FTLD-MND (Additional file 1: Figure S5b). These findings demonstrate that soluble pT153 levels differentiate A152T carriers from noncarriers, and may represent the underlying mechanism by which the A152T variant modulates disease risk.

## Discussion

The current study reveals very distinct neuropathological and behavioral characteristics observed in mice expressing the A152T risk variant in comparison to the pathogenic P301L mutation. In particular, while increased accumulation of hyperphosphorylated tau epitopes is noted with both mutations, hyperphosphorylated tau partitioned to the insoluble fraction in Tau^P301L^-AAV mice and remained soluble in Tau^A152T^-AAV mice. Our observation that the abnormal conformation detected by the MC1 antibody, which is believed to occur early in NFT formation [16, 24], is minimal in Tau^A152T^-AAV mice relative to the abundant accumulation in Tau^P301L^-AAV mice could indicate the A152T mutation inhibits tau aggregation by preventing adoption of the MC1 conformation. As our results indicate that A152T carriers exhibit increased soluble pT153, it is possible phosphorylation at T153 could inhibit the folding event required for the discontinuous MC1 epitope. Although transgenic mouse models expressing A152T tau independently generated by two different groups are reported to exhibit increased levels of MC1-positive tau species [19, 23], this is relative to nontransgenic littermates in one study [23], and mice overexpressing wild-type human tau in the second report [19]. Therefore the comparison performed in the current study between A152T and P301L, a pathogenic mutation prone to aggregate, very clearly demonstrates that while Tau^A152T^-AAV mice exhibit more MC1-positive labeling than nontransgenic mice injected with GFP-AAV (Fig. 1d, f), the amount of MC1-positive tau species present in Tau^A152T^-AAV relative to Tau^P301L^-AAV mice is negligible (Fig. 1e-f). In addition, consistent with our findings, transgenic A152T mice and *C.elegans* are reported to lack insoluble tau aggregates [5, 19, 21], implicating soluble tau species as a key mediator of neuronal dysfunction and hyperexcitability in A152T animals.

As increased inflammation is a common feature of tauopathies [2, 11, 12, 14, 18, 25] and was previously observed in Tau^P301L^-AAV mice at 6 months of age [8], we evaluated GFAP and IBA1 levels, which revealed that both markers are already significantly elevated in Tau^P301L^-AAV animals by 3 months of age. We also detected significant increases in GFAP at both the mRNA and protein level in Tau^A152T^-AAV mice, while IBA1 was slightly increased but did not reach significance. These results are consistent with observations in transgenic A152T mice [19, 23], and the demonstration that microgliosis is detected at 10 months of age [23] suggests that IBA1 would continue to increase in Tau^A152T^-AAV mice with age, which will need to be evaluated in future studies.

The behavioral assessment of Tau^A152T^-AAV and Tau^P301L^-AAV mice described in the current manuscript also revealed significant differences between models, most notably with the development of motor symptoms in Tau^A152T^-AAV animals. While the appearance of cognitive deficits is consistent across A152T models [19, 23], impaired motor performance is only observed in Tau^A152T^-AAV mice, most likely due to the presence of hyperphosphorylated tau in the spinal cord (Additional file 1: Figure S4). Moreover, the absence of a motor phenotype in the transgenic A152T models could potentially be attributed to the restriction of transgene expression to the forebrain in one model [19], and low transgene expression levels in the second model [23]. Given the flexibility of the SBT approach, these possibilities could easily be tested by either reducing viral titer and/or engineering a viral vector to direct Tau^A152T^-AAV expression to a specific cell-type or neuronal population.

Given the discovery that accumulation of soluble pT153-positive tau species differentiates A152T carriers from noncarriers, future studies are needed to assess whether this phospho-tau epitope is present in CSF and/or plasma and might be useful as a biomarker, as well as to determine if pT153 is detected in iPSCs from A152T carriers. Considering the recent advances in cryo-EM and mass-spectrometry techniques, it would also be intriguing to resolve the structure of tau filaments in A152T carriers and elucidate the extent to which the wild-type and A152T alleles contribute to pathology, in addition to the role of phosphorylation at T153 in aggregation. Given that our results indicate that pT153 species remain soluble in A152T carriers, this may suggest this phosphorylation event inhibits aggregation of A152T tau. While counterintuitive that reduced tau aggregation would be associated with increased tauopathy risk, neuronal loss and cognitive impairment were reported in the absence of insoluble tau deposition in an A152T transgenic mouse model [19], implicating soluble tau species in the neurodegenerative phenotype. In addition to supporting a greater focus and consideration of soluble tau in disease pathogenesis, given that A152T and P301L tau exhibit very different biochemical profiles in vivo, these findings further indicate that pathogenic tau mutations associated with FTDP-17 (such as P301L) may not accurately model Alzheimer’s tauopathy.

## Conclusions

In conclusion, we demonstrate that expression of P301L and A152T mutant tau result in very different neuropathological and behavioral phenotypes, with the A152T mutation driving accumulation of soluble hyperphosphorylated tau species and preventing an early conformational event linked to tau aggregation. In addition to cognitive deficits, A152T expression is also associated with motor impairment, the appearance of which is most likely determined by attaining a specific threshold of expression and/or hyperphosphorylated tau in the spinal cord. Our observation that soluble pT153-levels are specifically increased in Tau^A152T^-AAV mice and human A152T carriers provides additional insight into the consequences of A152T expression, and implicates phosphorylation of T153 and an altered solubility profile in the mechanism by which A152T influences disease risk. Moreover, as the A152T variant has been reported to modulate risk for AD, DLB and the FTD-spectrum disorders [9, 17], genetic modifiers most likely play a critical role in determining the resulting consequence of the mutation. Therefore our AAV-based model is uniquely-positioned to identify novel genetic variants that influence A152T-associated toxicity and phenotypic presentation.

## Additional file

Additional file 1: **Figure S1.** Accumulation of soluble, hyperphosphorylated tau in A152T-expressing mice. **Figure S2.** Increased tau phosphorylation in the sarkosyl-soluble S2 fraction in Tau^A152T^-AAV mice. **Figure S3.** Neurodegeneration observed in Tau^A152T^-AAV mice. **Figure S4.** Human tau expression is detected in the spinal cord of tau-AAV injected mice. **Figure S5.** Accumulation of soluble pT153 is specific to A152T carriers. (PDF 1530 kb)

## Abbreviations

AAV: Adeno-associated virus
AD: Alzheimer’s disease
CBD: Corticobasal degeneration
CRMP2: Collapsing response mediator protein-2
DLB: Dementia with Lewy bodies
EPM: Elevated plus maze
FTDP-17: Frontotemporal dementia and parkinsonism linked to chromosome 17
GFAP: Glial fibrillary acidic protein
GFP: Green fluorescent protein
MAPT: Microtubule associated protein tau
MSD: MesoScale discovery
NFT: Neurofibrillary tangle
OFA: Open field assay
PiD: Pick’s disease
PSP: Progressive supranuclear palsy
SBT: Somatic brain transgenesis
