# Supplementary material for: Enhanced phosphorylation of T153 in soluble tau is a defining biochemical feature of the A152T tau risk variant
Source: Acta Neuropathol Commun. 2019 Jan 23;7:10. doi: 10.1186/s40478-019-0661-2 (PMC6345061; doi:10.1186/s40478-019-0661-2)
Supplement: Supplementary file 1 — Figure S1. Accumulation of soluble, hyperphosphorylated tau in A152T-expressing mice. Figure S2. Increased tau phosphorylation in the sarkosyl-soluble S2 fraction in TauA152T-AAV mice. Figure S3. Neurodegeneration observed in TauA152T-AAV mice. Figure S4. Human tau expression is detected in the spinal cord of tau-AAV injected mice. Figure S5. Accumulation of soluble pT153 is specific to A152T carriers. (PDF 1530 kb) [file 40478_2019_661_MOESM1_ESM.pdf]

SUPPLEMENTARY DATA

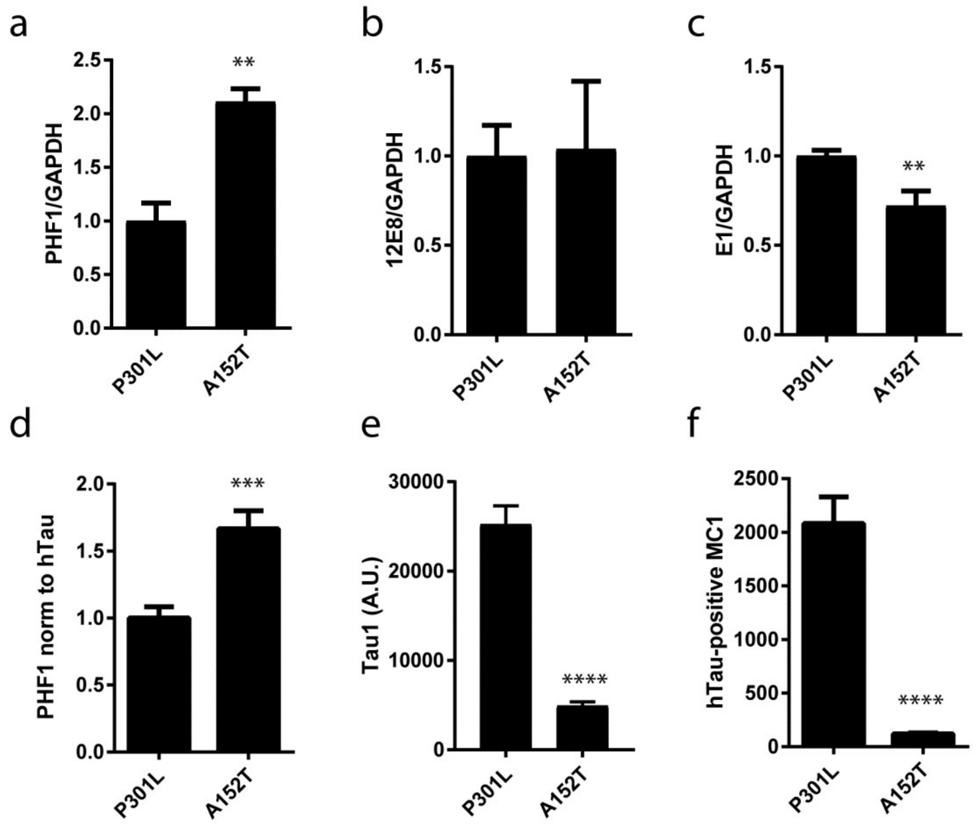

Figure S1. Accumulation of soluble, hyperphosphorylated tau in A152T-expressing mice. (**a-c**) Quantification of PHF1 (**a**; t=3.82, p=0.002), 12E8 (**b**; t=0.1, p=0.9) and E1 (**c**; t=3.048, p=0.006) levels in the S1 fraction by immunoblotting, with GAPDH used to control for protein loading. (**d**) Despite decreased total human tau protein expression, PHF1-positive human tau is significantly increased in Tau^{A152T}-AAV mice by MSD immunoassay (t=4.061, p=0.0006). (**e**) Consistent with an increase in tau phosphorylation in Tau^{A152T}-AAV mice, there is a significant reduction in Tau1 (unphosphorylated S195, 198, 199 and 202) levels by MSD immunoassay (t=10.01, p<0.0001). (**f**) Minimal MC1-positive human tau is detected in Tau^{A152T}-AAV relative to Tau^{P301L}-AAV mice (t=8.276, p<0.0001). **p<0.005, ***p<0.001, ****p<0.0001

SUPPLEMENTARY DATA

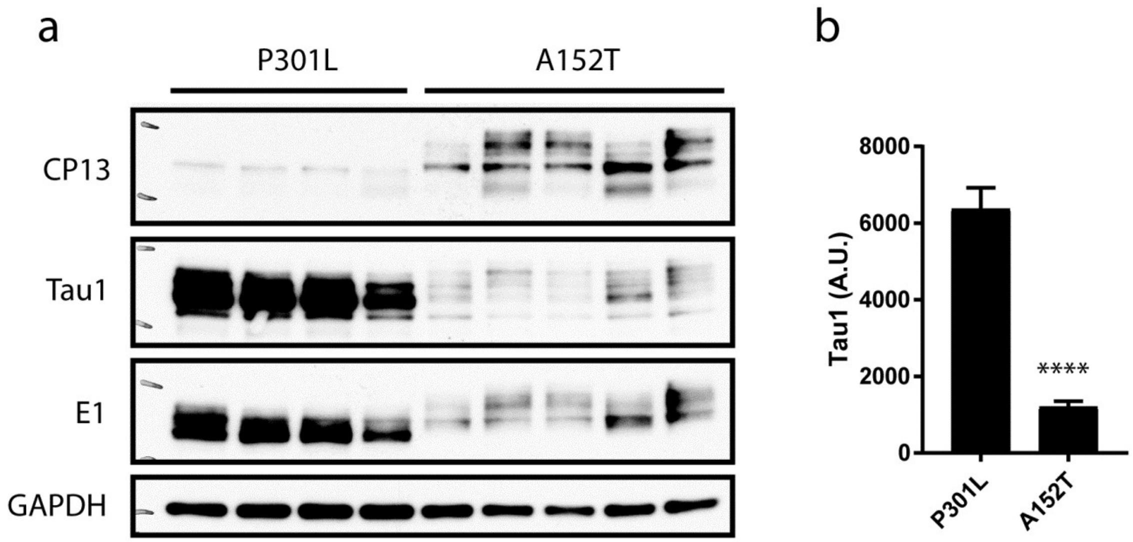

Figure S2. Increased tau phosphorylation in the sarkosyl-soluble S2 fraction in Tau^{A152T}-AAV mice. (**a**) Biochemical fractionation followed by immunoblotting of the sarkosyl-soluble S2 fraction demonstrates accumulation of phosphorylated tau in Tau^{A152T}-AAV mice, revealed by elevated CP13 (pS202) and reduced Tau1 (unphosphorylated S195/198/199/202). E1 levels are also lower in Tau^{A152T}-AAV mice, consistent with reduced human tau protein in the S2 fraction, and shifted to a higher molecular weight indicative of hyperphosphorylation. (**b**) Tau1 levels in the S2 fraction measured by MSD immunoassay are also significantly lower in Tau^{A152T}-AAV compared to Tau^{P301L}-AAV mice (t=9.431, p<0.0001). ****p<0.0001

SUPPLEMENTARY DATA

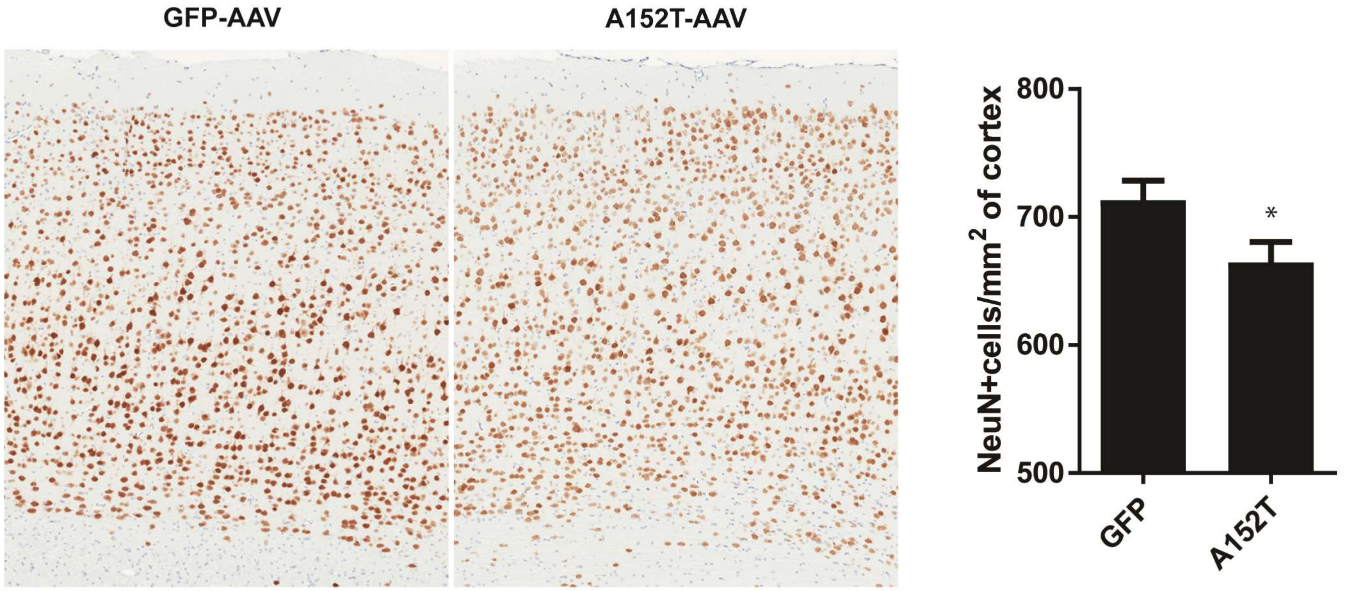

Figure S3. Neurodegeneration observed in Tau^{A152T}-AAV mice. Representative images of immunohistochemical analysis of NeuN in the cortex of GFP-AAV and Tau^{A152T}-AAV mice. The total number of NeuN-positive nuclei in the cortex was calculated, and divided by the total area to assess cortical neuron density, which was significantly reduced in Tau^{A152T}-AAV mice (t=2.183, p=0.04). *p<0.05

SUPPLEMENTARY DATA

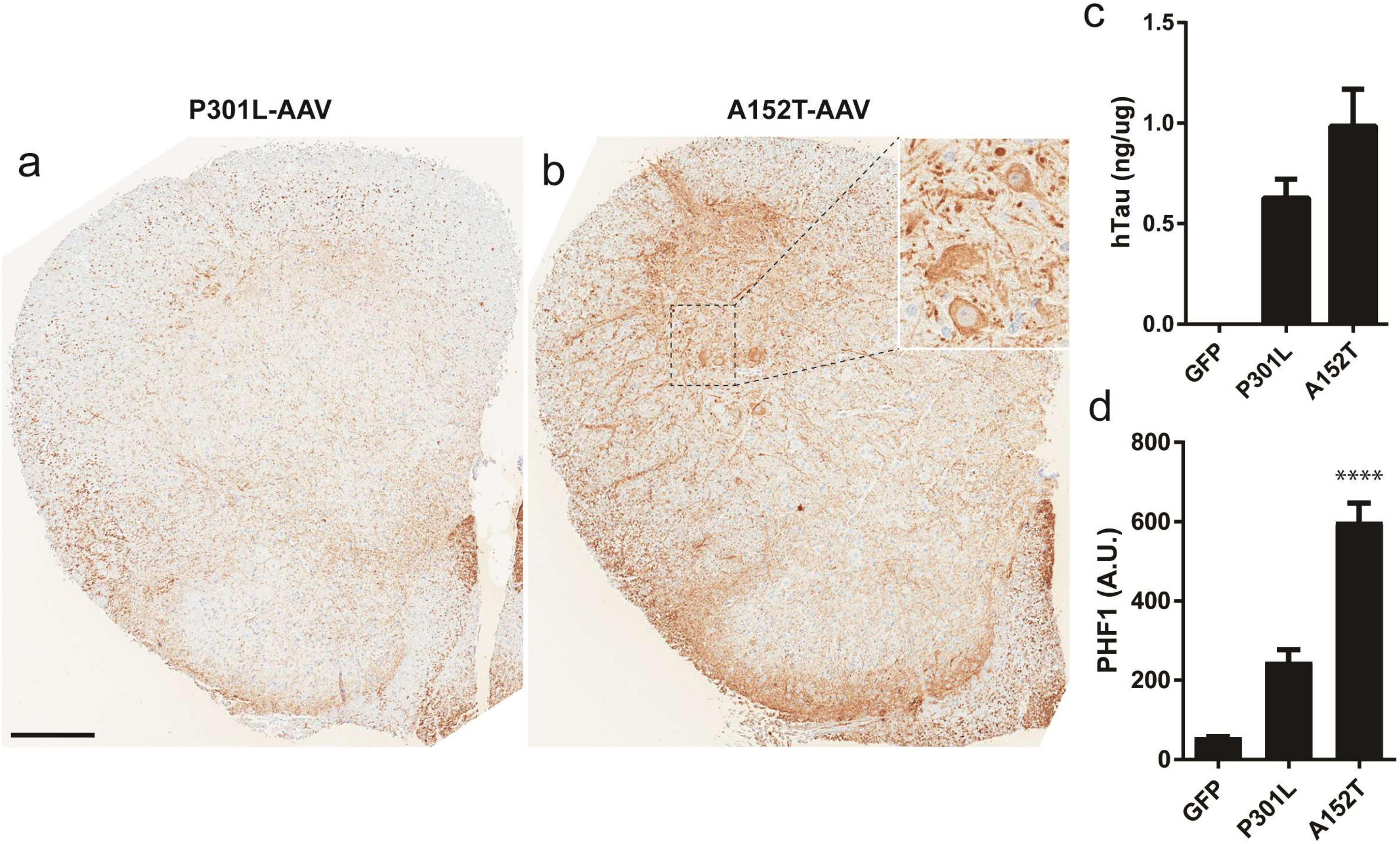

Figure S4. Human tau expression is detected in the spinal cord of tau-AAV injected mice. (a-b) Immunohistochemistry was used to evaluate human tau expression in spinal cord from Tau^{P301L}-AAV and Tau^{A152T}-AAV injected animals using E1 (human-tau specific antibody). (**c**) Total human tau levels were evaluated in spinal cord lysates by MSD immunoassay. Values are reported in ng/ug protein, based on a standard curve generated from full-length recombinant human tau. (**d**) Tau phosphorylated on the PHF1 epitope (pS396/404) in spinal cord lysates were measured by MSD immunoassay ($F=70.54$, $p<0.0001$). Scale bar 200μm.
**** $p<0.0001$

SUPPLEMENTARY DATA

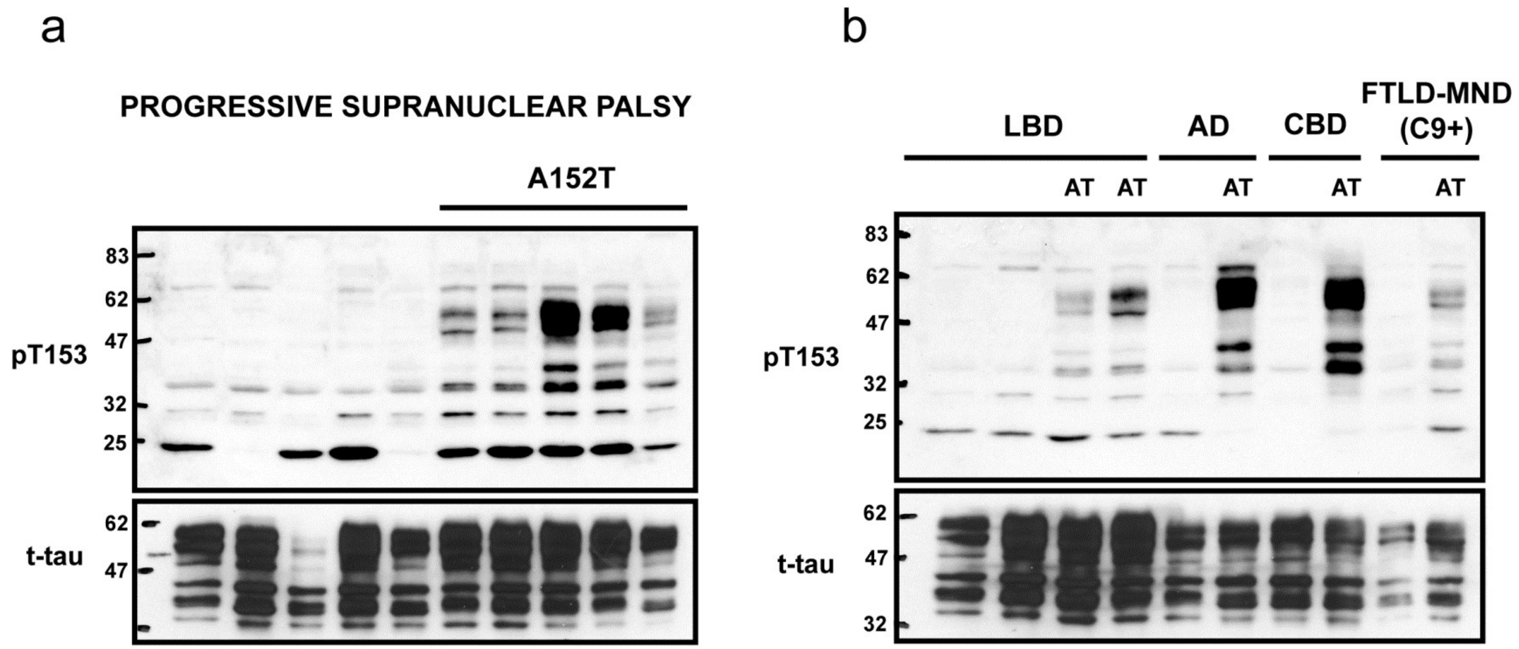

Figure S5. Accumulation of soluble pT153 is specific to A152T carriers. (a-b) Biochemical fractionation followed by immunoblotting of the soluble S1 fraction from frontal cortex reveals significant accumulation of pT153 in A152T carriers relative to noncarriers, independent of disease classification. LBD=Lewy body dementia; AD=Alzheimer's disease; CBD=corticobasal degeneration; FTLD-MND (C9+)=frontotemporal lobar degeneration with motor neuron disease associated with C9ORF72 mutation
